# The Therapeutic Potential of ADSC-Secreted LEFTY2 in Treating Alzheimer’s Disease

**DOI:** 10.3390/ijms26073382

**Published:** 2025-04-04

**Authors:** Wei Wuli, Hsueh-Hui Yang, Tzyy-Wen Chiou, Peng Yeong Woon, Yue-Xuan Xu, Cynthia Tjandra, Ivan Wijaya, Horng-Jyh Harn, Shinn-Zong Lin

**Affiliations:** 1 Bioinnovation Center, Buddhist Tzu Chi Medical Foundation, Hualien 97002, Taiwan; dionysus2316@gmail.com (W.W.); xuan01101994@gmail.com (Y.-X.X.); 2 Department of Medical Research, Hualien Tzu Chi Hospital, Buddhist Tzu Chi Medical Foundation, Hualien 97002, Taiwan; hhyang@tzuchi.com.tw; 3 Department of Life Science, National Dong Hwa University, Hualien 974301, Taiwan; twchiou@mail.ndhu.edu.tw; 4 Department of Molecular Biology and Human Genetics, Tzu Chi University, Hualien 97004, Taiwan; woon07@gms.tcu.edu.tw (P.Y.W.); 112727103@gms.tcu.edu.tw (C.T.); ivanwjy74@gmail.com (I.W.); 5 Department of Pathology, Hualien Tzu Chi Hospital, Hualien 97002, Taiwan; 6 Department of Neurology, Hualien Tzu Chi Hospital, Hualien 97002, Taiwan

**Keywords:** Alzheimer’s disease, Ts21 induced pluripotent stem cells, adipose derived mesenchymal stem cells, left–right determination factor 2, apolipoprotein E4

## Abstract

Adipose-derived mesenchymal stem cells (ADSCs) have exhibited promising therapeutic potential in Alzheimer’s disease (AD), although the underlying mechanisms remain poorly understood. Previously established Alzheimer’s disease neuron models derived from Ts21-induced pluripotent stem cells (Ts21-iPSCs) have been shown to exhibit progressive amyloid beta accumulation during neuronal differentiation. In this study, we employed a Transwell co-culture system to investigate the interaction between neurons derived from Ts21-iPSCs and ADSCs. Our findings revealed that co-culture with ADSCs significantly enhanced the survival rate of AD neurons. Proteomics analysis identified significant upregulation of left–right determination factor 2 (LEFTY2) protein in the co-culture medium. Supplementation with 2 nM LEFTY2 markedly improved the survival and growth of AD neurons. Furthermore, LEFTY2 effectively downregulates the expression of apolipoprotein E4 and amyloid beta 1–42, along with attenuating phosphorylated tau231 levels in AD neurons. These results suggest the potential of LEFTY2 as a promising therapeutic candidate for Alzheimer’s disease.

## 1. Introduction

Alzheimer’s disease (AD) is a progressive neurodegenerative disorder that afflicts over 55 million individuals globally [1]. Alzheimer’s disease (AD) is characterized by a range of pathological alterations, including synaptic dysfunction, mitochondrial impairment, and oxidative stress. Among these, our study focuses on three major hallmarks that are considered central to AD progression: the accumulation of amyloid beta (Aβ) plaques, the hyperphosphorylation of tau proteins, and chronic neuroinflammation.

Aβ is the main component of the amyloid plaques found in the brains of individuals diagnosed with AD [2]. Aβ is produced through the enzymatic cleavage of amyloid precursor protein (APP). The initial cleavage of full-length APP can occur via the β-secretase pathways [3]. These APP fragments are further cleaved by γ-secretase, culminating in the production of Aβ peptides, such as aggregation-prone Aβ (Aβ42). These peptides can subsequently form toxic Aβ plaques in the brain [4].

Tau, a microtubule-associated protein, is essential for maintaining microtubule stability in neurons. In AD, Tau undergoes abnormal hyperphosphorylation, resulting in its dissociation from microtubules and subsequent aggregation into neurofibrillary tangles [1]. These Tau aggregates disrupt normal neuronal function and ultimately contribute to neurodegeneration. Concurrently, neuroinflammation is one of the hallmarks of AD pathology. In response to the accumulation of Aβ and Tau, activated microglia and astrocytes release pro-inflammatory cytokines [5,6]. This persistent inflammatory response exacerbates neuronal injury, establishing a detrimental cycle that accelerates the progression of AD.

Presenilin 1 (PSEN1) and amyloid precursor protein (APP) are well-established genetic factors associated with early-onset familial Alzheimer’s disease (EOFAD), whereas apolipoprotein E4 (APOE4) is a recognized genetic risk factor for sporadic late-onset Alzheimer’s disease (LOAD). The APOE4 allele, in particular, has been shown to contribute to the disease, especially when inherited from both parents. The PSEN1 gene encodes proteins involved in the processing of APP. Mutations in these genes can lead to the overproduction of Aβ and associated plaques in the brain. APP is a protein processed to produce Aβ and other fragments. Mutations in the APP gene can also increase the risk of AD by altering the production or processing of Aβ [7].

Ts21-induced pluripotent stem cells demonstrate a remarkable capacity to differentiate into neural cells and produce amyloid protein upon maturation. The presence of an extra chromosome 21 in Ts21-induced pluripotent stem cells (Ts21-iPSCs) leads to the overexpression of the APP gene, resulting in increased production and accumulation of Aβ, thereby accelerating the formation of amyloid plaques. Numerous studies have employed Ts21-iPSCs as a valuable tool in AD research [8].

In addition to conventional drug therapies, adipose mesenchymal stem cells (ADSCs) are emerging as promising candidates for treating AD due to their multifaceted therapeutic potential. ADSCs exhibit potent anti-inflammatory properties and promote the regeneration of cerebral blood vessels and neurons. These regenerative capabilities may contribute to improving cognitive function and alleviating dementia symptoms [9]. Moreover, ADSCs secrete diverse growth factors that can stimulate the proliferation of endogenous neural stem cells [10], suppress the expression of pro-inflammatory cytokines, and enhance the production of anti-inflammatory cytokines [11]. Consequently, the therapeutic potential of ADSC-derived exosomes for various diseases, including AD, has earned an increase in attention in recent years [12].

An indirect co-culture system involving ADSCs [13] was established to investigate the potential bioactive molecules secreted by ADSCs. Proteomic analysis of the culture medium revealed a significant upregulation of left–right determination factor 2 (LEFTY2) in the co-culture of ADSCs and Ts21 neurons. LEFTY2, also known as TGF-β4, is well-established for its role in regulating embryonic stem cell differentiation and self-renewal [14]. Additionally, LEFTY2 possesses the ability to inhibit TGF-β signaling [15]. However, the specific role of LEFTY2 in AD remains an area of active research. Consequently, this study aims to delve deeper into the potential of LEFTY2 in mitigating amyloid formation and to elucidate its influence on AD-related genes and molecular pathways.

## 2. Results

### 2.1. Co-Culture of Ts21 Neurons with ADSCs Significantly Enhances the Population of TS21 Neurons and Downregulates the Expression of the APOE Gene and Protein

**Figure 1:**
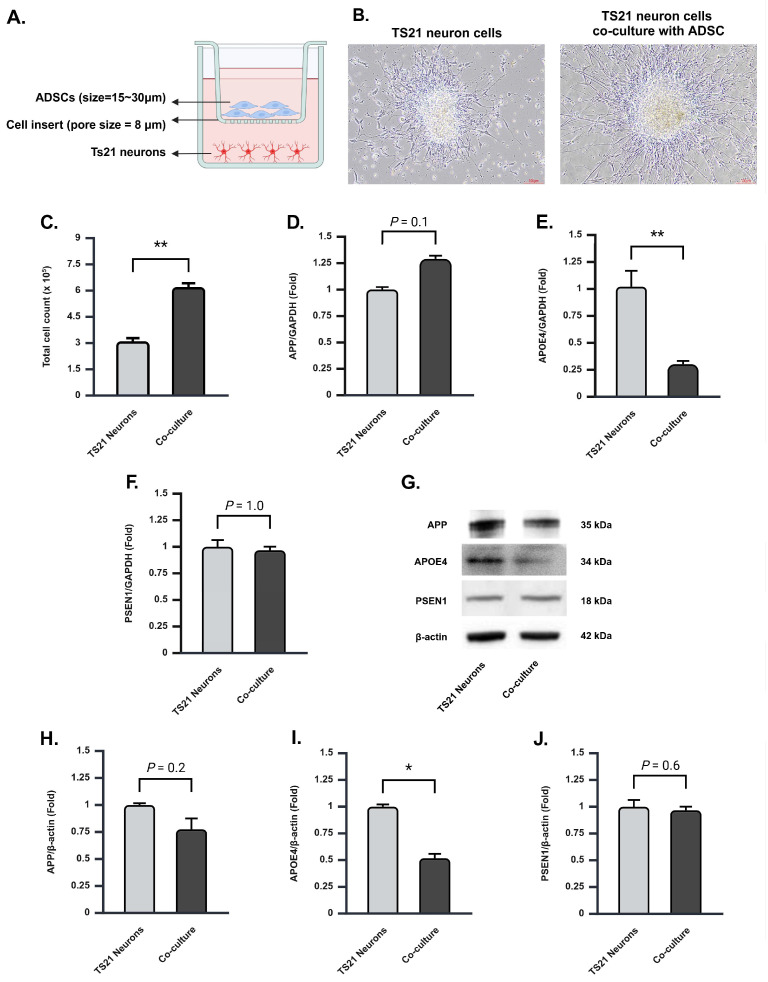
Co-culture of Ts21 neurons with ADSCs enhances the population of TS21 neurons and downregulates the expression of APOE gene and protein. (**A**) Schematic representation of ADSCs and Ts21 neuron co-culture system. (**B**) Microscopic image of representative Ts21 neuron cells (left) compared to Ts21 neuron cells co-cultured with ADSCs (right). Scale bar: 100 μm. (**C**) The average number of Ts21 neurons in the single and co-cultured groups evaluated using cell viability analysis (single = 310,000 ± 18,027, co-cultured = 620,000 ± 21,794), *n* = 3 for each group. (**D**–**F**) Real-time PCR analysis of (**D**) APP, (**E**) APOE4, and (**F**) PSEN1 in Ts21 neurons single and co-culture groups. APOE4 gene expression significantly decreased in Ts21 neurons following co-culture (single = 1.02 ± 0.15, co-culture = 0.30 ± 0.03). In contrast, no significant reduction was observed in APP (single = 1 ± 0.02, co-culture = 1.29 ± 0.03) and PSEN1 (single = 1 ± 0.06, co-culture = 0.97 ± 0.03) genes, *n* = 3 for each group. (**G**) Representative blot of APP, PSEN1, and APOE4 in Ts21 neuron single and co-culture group. (**H**–**J**) Quantitative Western blot analysis of (**H**) APP, (**I**) APOE4, and (**J**) PSEN1 in Ts21 neuron single and co-culture group. APOE4 protein expression significantly decreased in Ts21 neurons following co-culture (single = 1 ± 0.02, co-culture = 0.52 ± 0.04). In contrast, no significant reduction was observed in APP (single = 1 ± 0.02, co-culture = 0.78 ± 0.10) and PSEN1 (single = 1 ± 0.06, co-culture = 0.97 ± 0.03) proteins, *n* = 3 for APP and PSEN1, *n* = 4 for APOE4. Values are mean ± SEM; * *p* < 0.05. ** *p* < 0.01 by Student’s *t*-tests.

We co-cultured Ts21 neurons with ADSCs in a Transwell co-culture system employing an 8 μm cell insert. This configuration effectively prevented direct cell–cell contact (Figure 1A). We performed patch-clamp analysis to determine whether neurons derived from Ts21 iPSCs develop an Alzheimer’s disease (AD)-like phenotype compared to those derived from normal iPSCs. Our results revealed significant differences in the electrical properties of Ts21 neurons, including prolonged depolarization and delayed repolarization, relative to normal iPSC-derived neurons. These findings indicate that Ts21 neurons exhibit electrophysiological abnormalities associated with AD-like pathology (Supplementary Figure S1). Microscopic observation revealed a statistically significant increase in the number of Ts21 neurons within the co-culture group compared to the individual culture group (Figure 1B and Supplementary Figure S2). Cell viability analysis further corroborated this finding, demonstrating a two-fold increase in the number of surviving Ts21 neurons in the co-culture system (620,000 ± 21,794) (Figure 1C).

As a model for AD research, Ts21-iPSCs have been shown to exhibit increased expression of AD-associated genes, including APP, PSEN1, and APOE4. These genes were analyzed following the co-culture experiment. Real-time PCR analysis revealed a significant reduction in APOE4 gene expression in Ts21 neurons (Figure 1E). However, no such reduction was observed in APP and PSEN1 genes (Figure 1D,F). Conversely, co-culture conditions led to an increase in APOE3 expression (Supplementary Figure S3). Western blot analysis was employed to assess APP, PSEN1, and APOE4 protein expression. Consistent with real-time PCR results, a significant decrease in APOE4 protein was observed (Figure 1G,I, Supplementary Figure S4), while the expression of APP and PSEN1 proteins remained relatively unchanged (Figure 1G,H,J, Supplementary Figure S4). Our findings collectively suggest that the co-culture of Ts21 neurons with ADSCs significantly enhances the population of TS21 neurons and downregulates the expression of the APOE gene and protein, suggesting a potential therapeutic benefit for AD.

### 2.2. The Co-Culture of TS21 Neurons and ADSCs Significantly Elevates LEFTY2 Protein

**Figure 2:**
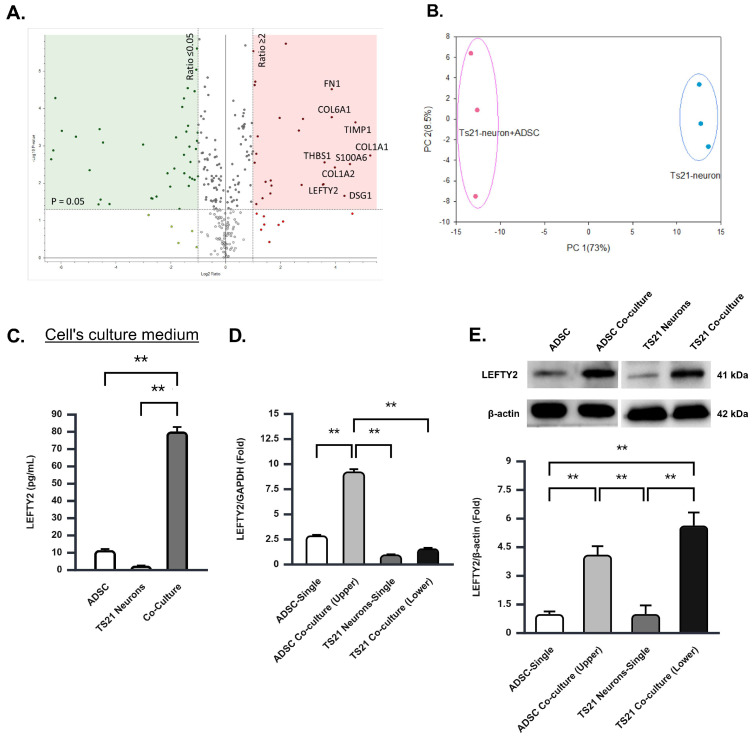
Co-culture of Ts21 neurons with ADSCs resulted in elevated levels of LEFTY2 protein, primarily originating from ADSCS secretions. (**A**) Volcano plot of differentially expressed proteins quantified using LC–MS/MS. The vertical dotted lines mark twofold change, and the horizontal dotted line represents cut-off (*p* = 0.05). Proteins that showed fold changes greater than 2 or less than 0.5 and *p* < 0.05 are considered as up- or downregulated and marked in red and green, respectively. The grey dots are considered as having no significant change. (**B**) Principal component analysis (PCA) from ratio values of the differential expressed proteins in Ts21 neuron culture medium with and without ADSCs. The PCA plot indicated clear proteomics profile differences between them. The red plots represent the co-culture group, and the blue plots represent the control group. (**C**) LEFTY2 levels in the medium of single-cultured ADSCs, Ts21 neurons, and co-cultured cells were evaluated using ELISA. LEFTY2 levels are significantly increased in the co-culture medium (ADSCs single = 11.59 ± 0.58, Ts21 single = 0.14 ± 0.01, co-culture = 1.37 ± 0.01), *n* = 3 for each group. (**D**) Real-time PCR analysis of LEFTY2 mRNA in ADSCs and Ts21 neurons following single or co-culture. LEFTY2 gene expression increased significantly in ADSCs upon co-culture, but no significant changes were observed in ADSCs in single culture or in Ts21 neurons under both culture conditions. (ADSCs single 2.91 ± 0.04=, Ts21 neurons single = 1.00 ± 0.03, ADSCs co-culture = 9.26 ± 0.23, Ts21 neurons co-culture = 1.62 ± 0.04), *n* = 3 for each group. (**E**) Representative blot (top) and quantitative Western blot analysis (bottom) of LEFTY2 in ADSCs and Ts21 under single and co-culture conditions. LEFTY2 protein expression significantly increased in both ADSCs and Ts21 neurons following co-culture (ADSCs single = 1.00 ± 0.13, Ts21 neurons single = 1.00 ± 0.45, ADSCs co-culture = 4.10 ± 0.45, Ts21 neurons co-culture = 5.63 ± 0.69), *n* = 4. Values are mean ± SEM; ** *p* < 0.01 by one-way ANOVA followed by Tukey post hoc test.

We conducted proteomic analysis of the cell culture medium to elucidate the underlying mechanisms responsible for enhanced survival rate and downregulation of APOE4 gene and protein expression. The analysis identified the presence of specific proteins within the co-culture group, including LEFTY2, S100-A6, Cathepsin B, Lumican, Thrombospondin-1, and transforming growth factor-beta-induced protein ig-h3 (Figure 2A). Principal component analysis was employed to compare the proteomic profiles of the cell culture medium derived from the Ts21 neuron co-culture with ADSCs and Ts21 neurons-only groups. The results demonstrated distinct patterns between the two groups, confirming the association between the co-culture environment and the secreted proteome (Figure 2B). Enzyme-linked immunosorbent assay (ELISA) analysis confirmed that the expression pattern of LEFTY2 exhibited the most significant concordance with the proteomic analysis results. The analysis revealed a statistically significant increase in the level of LEFTY2 protein in the co-culture group compared to either of the single cell culture groups (Figure 2C).

### 2.3. Upregulation of LEFTY2 Protein in the Co-Culture Is Primarily Driven by ADSCS Secretion

To identify the cellular origin of LEFTY2 protein, we performed real-time PCR analysis to detect LEFTY2 expression in both ADSCs and Ts21 neurons. Our findings revealed a significant upregulation of LEFTY2 mRNA expression in ADSCs upon co-culture, whereas no such increase was observed in ADSCs in single culture or in Ts21 neurons under both culture conditions. (Figure 2D). Subsequent Western blot analysis revealed a significant increase in LEFTY2 protein expression in both ADSCs and Ts21 neurons under co-culture conditions. In contrast, LEFTY2 protein expression remained unchanged when both cells were cultured independently (Figure 2E, Supplementary Figure S5). These findings indicate that the elevated levels of LEFTY2 within the co-culture system are primarily derived from ADSCs and subsequently taken up by Ts21 neurons. Our findings suggest that LEFTY2, a protein secreted by ADSCs, may serve as a key modulator in promoting the proliferation of Ts21 neurons.

### 2.4. LEFTY2 Supplementation Stimulates Neuronal Growth and Upregulates Synaptic Protein Expression in Ts21 Neurons

**Figure 3:**
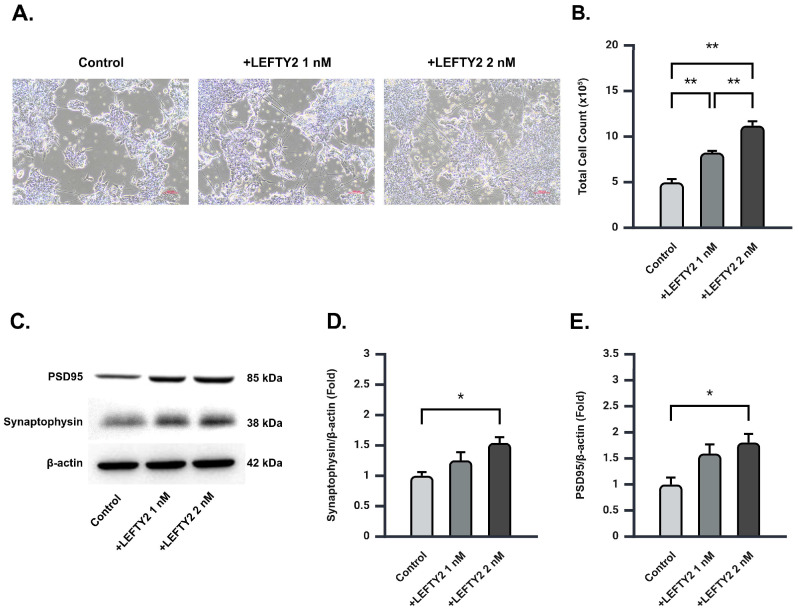
LEFTY2 enhances neuronal growth and synaptic protein expression in Ts21 neurons. (**A**) Microscopic image of a representative Ts21 neuron cell supplemented with 0 nM (left), 1 nM (middle), and 2 nM (right) of LEFTY2. Scale bar: 100 μm. (**B**) The average number of Ts21 neurons following supplementation of 0 nM, 1 nM, and 2 nM of LEFTY2. A dose-dependent increase in cell number was observed (control = 4.97 × 10^5^ ± 0.35 × 10^5^, 1 nM LEFTY2 = 8.23 × 10^5^ ± 0.19 × 10^5^, 2 nM LEFTY2 = 11.19 × 10^5^ ± 0.49 × 10^5^), *n* = 3 for each group. (**C**) Representative blot of PSD95 and synaptophysin in Ts21 neurons following supplementation of 0 nM, 1 nM, and 2 nM of LEFTY2. (**D**,**E**) Quantitative Western blot analysis of (**D**) PSD95 and (**E**) synaptophysin in Ts21 neurons following supplementation of 0 nM, 1 nM, and 2 nM of LEFTY2. A dose-dependent increase in PSD95 (control = 1.00 ± 0.06, 1 nM LEFTY2 = 1.25 ± 0.14, 2 nM LEFTY2 = 1.54 ± 0.09) and synaptophysin (control = 1.00 ± 0.13, 1 nM LEFTY2 = 1.59 ± 0.18, 2 nM LEFTY2 = 1.81 ± 0.17) protein level was observed, *n* = 3 for each group. Values are mean ± SEM; * *p* < 0.05. ** *p* < 0.01 by one-way ANOVA followed by Tukey post hoc test.

To better understand the effect of LEFTY2 secreted by ADSCs on Ts21 neurons, we supplemented Ts21 neuron cultures with 0 nM, 0.5 nM, 1 nM, 1.5 nM, and 2 nM of LEFTY2 recombinant protein. Microscopic observation revealed a significant increase in the number of nerve cells post-LEFTY2 supplementation (Figure 3A and Supplementary Figure S6). This observation was verified by cell viability assays, which confirmed an elevated number of viable neurons post-treatment (Figure 3B and Supplementary Figure S6). However, supplementation with 0.5 nM, 1 nM, and 1.5 nM of LEFTY2 showed no significant differences when compared to each other (Supplementary Figure S6), while 2 nM exhibited the most distinct effect.

Synaptophysin (SYN) and postsynaptic density protein 95 (PSD-95) are essential proteins in neuronal development and synapse formation. Previous studies have demonstrated significantly reduced SYN levels in AD and Down syndrome patients, with increased SYN correlating positively with improved memory [16,17]. Moreover, PSD-95 has been shown to protect synapses from Aβ toxicity [18]. Following 2 nM supplementation of LEFTY2, we observed a significant upregulation of SYN and PSD-95 protein expression in Ts21 neurons (Figure 3C–E, Supplementary Figure S7). Our findings suggest that LEFTY2 plays a role in neuroprotection by enhancing synaptic protein expression, thereby promoting neuronal growth.

### 2.5. LEFTY2 Supplementation Attenuates the Expression of Amyloid Beta 1–42 in Ts21 Neurons

**Figure 4:**
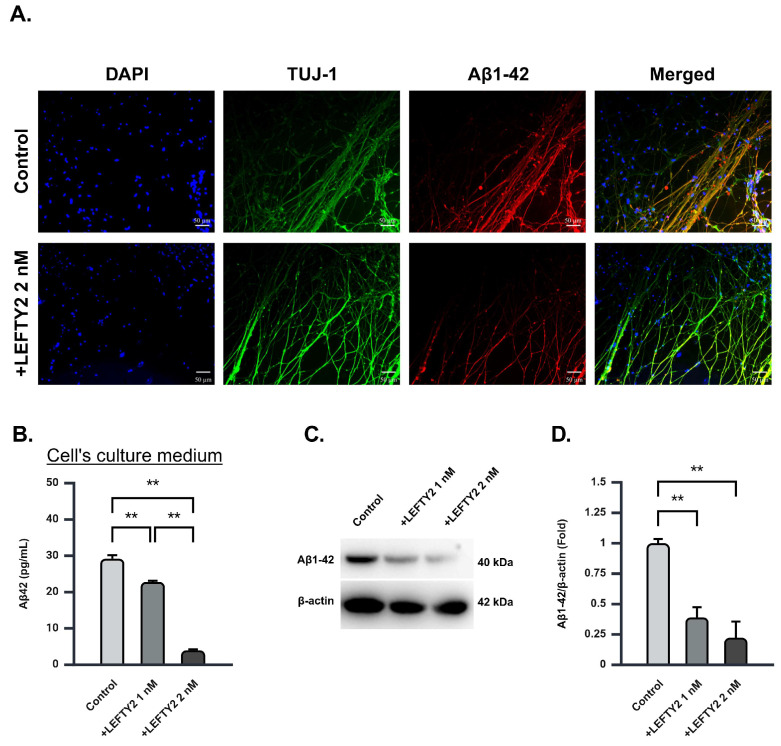
LEFTY2 supplementation attenuates Aβ1-42 in Ts21 neurons. (**A**) Fluorescence microscopy analysis of Aβ1-42 in Ts21 neurons following supplementation of 2 nM of LEFTY2. Cells were dispersed, fixed, and stained for DAPI, TUJ-1, and Aβ1-42 (DAPI, TUJ-1, and Aβ1-42 are indicated by blue, green, and red staining, respectively). Scale bar: 50 μm. (**B**) Aβ1-42 levels in Ts21 neuron culture medium following 0 nM, 1 nM, and 2 nM of LEFTY2 supplementation evaluated using ELISA. A dose-dependent decrease in Aβ1-42 levels in culture medium was observed (control = 3.70 ± 0.06, 1 nM LEFTY2 = 3.53 ± 0.20, 2 nM LEFTY2 = 3.30 ± 0.25), *n* = 3 for each group. (**C**) Representative blot of Aβ1-42 in Ts21 neurons following supplementation of 0 nM, 1 nM, and 2 nM of LEFTY2. (**D**) Quantitative Western blot analysis of Aβ1-42 in Ts21 neurons following supplementation of 0 nM, 1 nM, and 2 nM of LEFTY2. A dose-dependent decrease in Aβ1-42 protein level was observed (control = 1.00 ± 0.04, 1 nM LEFTY2 = 0.39 ± 0.08, 2 nM LEFTY2 = 0.22 ± 0.13), *n* = 3 for each group. Values are mean ± SEM; ** *p* < 0.01 by one-way ANOVA followed by Tukey post hoc test.

During differentiation, Ts21 neurons progressively generate amyloid proteins, notably amyloid beta 1–42 (Aβ1-42), a primary component of cerebral amyloid plaques [19]. To assess Aβ1-42 levels in Ts21 neurons, we performed immunostaining following treatment with 0 nM and 2 nM of LEFTY2. Fluorescence microscopy analysis revealed a significant reduction in Aβ1-42 red fluorescence intensity upon treatment with 2 nM LEFTY2 (Figure 4A). Furthermore, DAPI staining indicated an increase in the number of nuclei in LEFTY2-treated cells compared to the control group, suggesting enhanced cell viability (Supplementary Figure S8). To further investigate the effects of LEFTY2 supplementation on Aβ1-42 levels in Ts21 neurons, we expanded the analysis by treating the cells with 0 nM, 1 nM, and 2 nM of LEFTY2, as previously described. ELISA analysis revealed a significant change in Aβ1-42 concentration within the culture medium (Figure 4B). Subsequently, Western blot analysis confirmed a reduction in Aβ1-42 concentration within Ts21 neurons (Figure 4C,D, Supplementary Figure S9).

### 2.6. LEFTY2 Strongly Binds to APOE4 and Downregulates Its Protein Expression in Ts21 Neurons

**Figure 5:**
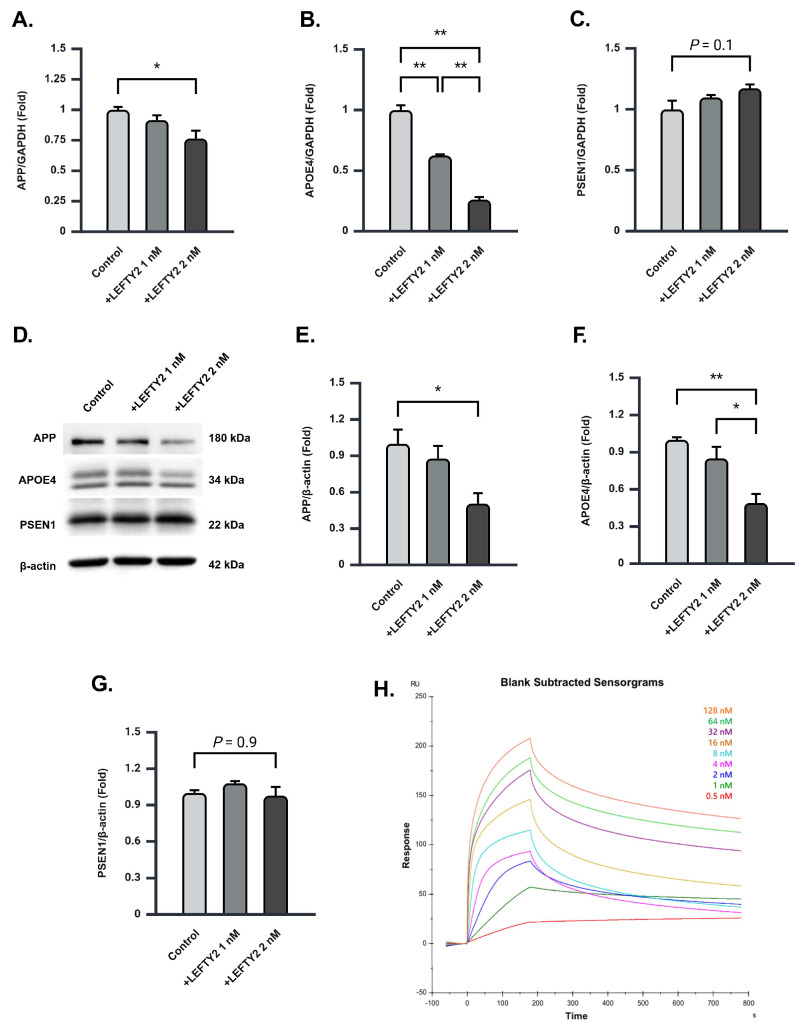
LEFTY2 binds to APOE4 and downregulates its protein expression in Ts21 neurons. (**A**–**C**) Real-time PCR analysis of (**A**) APP, (**B**) APOE4, and (**C**) PSEN1 in Ts21 neurons following supplementation of 0 nM, 1 nM, and 2 nM of LEFTY2. A dose-dependent decrease in APOE4 gene expression following LEFTY2 supplementation was observed (control = 1.00 ± 0.04, 1 nM LEFTY2 = 0.62 ± 0.01, 2 nM LEFTY2 = 0.26 ± 0.02). In contrast, only 2 nM LEFTY2 supplementation significantly reduced APP expression (control = 1.00 ± 0.02, 1 nM LEFTY2 = 0.92 ± 0.04, 2 nM LEFTY2 = 0.76 ± 0.06), while no significant changes were observed in PSEN1 expression (control = 0.99 ± 0.07, 1 nM LEFTY2 = 1.10 ± 0.02, 2 nM LEFTY2 = 1.17 ± 0.03) genes, *n* = 3 for each group. (**D**) Representative blot of APP, APOE4, and PSEN1 in Ts21 neurons following supplementation of 0 nM, 1 nM, and 2 nM of LEFTY2. (**E**–**G**) Quantitative Western blot analysis of (**E**) APP, (**F**) APOE4, and (**G**) PSEN1 in Ts21 neurons following supplementation of 0 nM, 1 nM, and 2 nM of LEFTY2. A dose-dependent decrease in APOE4 protein expression following LEFTY2 supplementation was observed (control = 1.00 ± 0.02, 1 nM LEFTY2 = 0.85 ± 0.09, 2 nM LEFTY2 = 0.49 ± 0.07). APP protein levels were significantly decreased only at 2 nM LEFTY2 supplementation (control = 1.00 ± 0.11, 1 nM LEFTY2 = 0.88 ± 0.10, 2 nM LEFTY2 = 0.51 ± 0.08). Conversely, no significant reduction was observed in PSEN1 protein expression (control = 1.00 ± 0.02, 1 nM LEFTY2 = 1.08 ± 0.02, 2 nM LEFTY2 = 0.97 ± 0.07), *n* = 3 for each group. (**H**) Multi-cycle kinetic analysis of human LEFTY2 to human ApoE4. No high-binding affinity was observed between APOE4 and LEFTY at concentrations ranging from 0.5 nM to 1 nM. However, a high binding affinity was detected for LEFTY2 concentrations starting at 2 nM with this affinity increasing proportionally to the LEFTY2 concentration. Values are mean ± SEM; * *p* < 0.05. ** *p* < 0.01 by one-way ANOVA followed by Tukey post hoc test.

Co-culture of Ts21 neurons with ADSCs resulted in the downregulation of AD-related genes, specifically APOE4 (Figure 1). To further investigate whether the downregulation of AD-related genes following co-culture is mediated by LEFTY2 protein, quantitative real-time polymerase chain reaction (RT-qPCR) analysis was conducted on APP, PSEN1, and APOE4 *genes*. A dose-dependent decrease in APOE4 gene expression within Ts21 neurons was observed upon increasing concentrations of LEFTY2 supplementation (Figure 5B). In contrast, APP expression was significantly altered only at 2 nM LEFTY2 supplementation, while no significant changes were detected in PSEN1 expression (Figure 5A,C). Our criteria for significant expression changes require at least a 0.5-fold decrease or increase. Although APP expression exhibited a statistically significant reduction at 2 nM LEFTY2 supplementation, the decrease did not reach the 0.5-fold threshold and was therefore not considered substantially significant. Additionally, our qPCR analysis revealed a dose-dependent increase in APOE3 gene expression with higher concentrations of LEFTY2 supplementation (Supplementary Figure S10). Western blot analysis was conducted to investigate whether LEFTY2 supplementation could also influence the expression of APP, PSEN1, and APOE4 proteins. The results demonstrated a significant downregulation of APP and APOE4 expression upon supplementation with 2 nM LEFTY2 (Figure 5D–F), while the expression of PSEN1 protein remained unchanged (Figure 5D,G).

Protein–protein binding analysis was performed with surface plasmon resonance to further understand the specific mechanism underlying the downregulation of the APOE4 gene and protein expression by LEFTY2 supplementation. The results predicted a high-affinity interaction between LEFTY2 and APOE4, starting from 2 nM of LEFTY2, providing a mechanistic basis for the observed effect (Figure 5H). Our findings collectively suggest that LEFTY2 may exert a protective effect by inhibiting the expression of APOE4, a key protein involved in Aβ1-42 production, which may attenuate neuronal Aβ toxicity and subsequent amyloid plaque formation.

### 2.7. LEFTY2 Supplementation Attenuates Phosphorylated Tau-231 Levels

**Figure 6:**
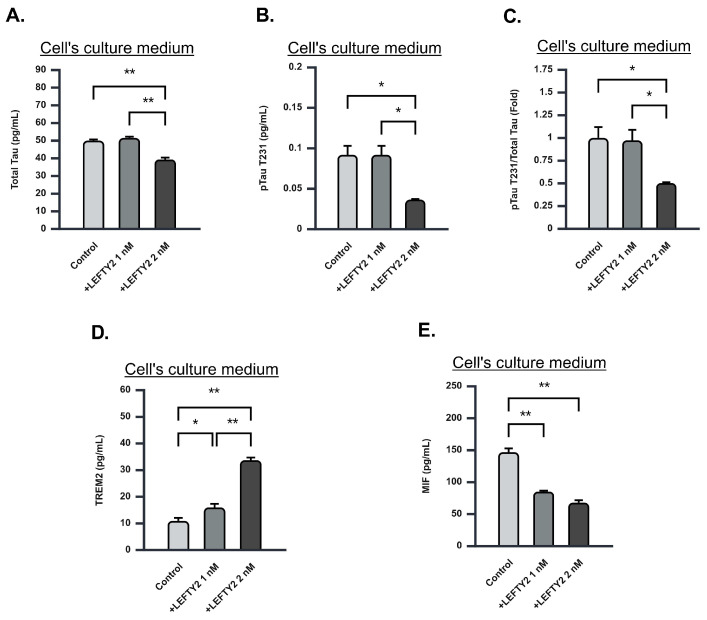
LEFTY2 supplementation attenuates phosphorylated Tau-231 levels and neuroinflammation. (**A**) Total Tau levels in Ts21 neuron culture medium following 0 nM, 1 nM, and 2 nM of LEFTY2 supplementation evaluated using ELISA. Supplementation with 2 nM LEFTY2 significantly reduced total Tau levels in the culture medium. (Control = 50.05 ± 0.63, 1 nM LEFTY2 = 51.57 ± 0.73, 2 nM LEFTY2 = 39.40 ± 1.06), *n* = 3 for each group. (**B**) Phosphorylated Tau-231 levels in Ts21 neuron culture medium following 0 nM, 1 nM, and 2 nM of LEFTY2 supplementation evaluated using ELISA. pTau-231 levels were significantly decreased in the culture medium following supplementation with 2 nM LEFTY2. (Control = 0.09 ± 0.01, 1 nM LEFTY2 = 0.09 ± 0.01, 2 nM LEFTY2 = 0.04 ± 0.01), *n* = 3 for each group. (**C**) Phosphorylated Tau-231 to total Tau fold ratio following 0 nM, 1 nM, and 2 nM of LEFTY2 supplementation evaluated using ELISA. pTau-231 levels were significantly decreased in the culture medium following supplementation with 2 nM LEFTY2. (Control = 1.00 ± 0.12, 1 nM LEFTY2 = 0.97 ± 0.12, 2 nM LEFTY2 = 0.50 ± 0.01), *n* = 3 for each group. (**D**) Trem2 levels in Ts21 neuron culture medium following 0 nM, 1 nM, and 2 nM of LEFTY2 supplementation evaluated using ELISA. Supplementation with 1 nM and 2 nM LEFTY2 significantly enhanced Trem2 levels in the culture medium. (Control = 10.95 ± 1.08, 1 nM LEFTY2 = 16.01 ± 1.30, 2 nM LEFTY2 = 33.73 ± 0.97), *n* = 3 for each group. (**E**) MIF levels in Ts21 neuron culture medium following 0 nM, 1 nM, and 2 nM of LEFTY2 supplementation evaluated using ELISA. MIF levels in the culture medium were significantly reduced following supplementation with 1 nM and 2 nM LEFTY2. (Control = 147.02 ± 5.77, 1 nM LEFTY2 = 85.31 ± 1.50, 2 nM LEFTY2 = 68.06 ± 3.79), *n* = 3 for each group. Values are mean ± SEM; * *p* < 0.05. ** *p* < 0.01 by one-way ANOVA followed by Tukey post hoc test.

To assess the effect of LEFTY2 on Tau pathology, we quantified total Tau and phosphorylated Tau (pTau) levels in the cell culture medium using ELISA. A 2 nM amount of LEFTY2 supplementation resulted in a significant reduction in total Tau levels compared to the control group (Figure 6A). Additionally, pTau at the T231 site exhibited a significant decrease following LEFTY2 treatment (Figure 6B,C). In contrast, no significant alterations were detected in pTau levels at the T181 and S199 sites (Supplementary Figure S11). Our findings suggest that LEFTY2 selectively modulates specific phosphorylation sites on Tau, potentially influencing Tau-related neurotoxicity.

### 2.8. LEFTY2 Supplementation Enhances Triggering Receptor Expressed on Myeloid Cells 2 (TREM2) Expression While Downregulating Macrophage Migration Inhibitory Factor (MIF) Expression

Increased levels of pTau are often associated with neuroinflammation. To further investigate the effects of LEFTY2 on Tau pathology, we measured levels of macrophage migration inhibitory factor (MIF) and triggering receptor expressed on myeloid cells 2 (TREM2) using ELISA in the cell culture medium following LEFTY2 supplementation. Supplementation with 1 nM and 2 nM of LEFTY2 similarly increased TREM2 levels, suggesting enhanced neuroprotective responses (Figure 6D). Conversely, LEFTY2 supplementation resulted in a significant downregulation of MIF levels in the culture medium (Figure 6E), indicating that LEFTY2 effectively suppresses MIF expression extracellularly and intracellularly. Our findings suggest that LEFTY2 not only modulates cytokine secretion but also influences intracellular cytokine dynamics, potentially contributing to an anti-inflammatory and neuroprotective environment.

## 3. Discussion

This study reveals for the first time the multifaceted effects of LEFTY2 protein in the co-culture of ADSCs and Ts21 neurons, underscoring its promising therapeutic potential for neuroprotection and neurodegenerative diseases. LEFTY2, predominantly secreted by ADSCs, is abundantly present in the co-culture environment, suggesting that ADSCs exert their influence on peripheral nerve cells not only through direct cell–cell interactions but also via the secretion of bioactive molecules. Moreover, LEFTY2-mediated upregulation of SYN and PSD-95 proteins may contribute to the promotion of nerve cell growth and synaptic stability, ultimately leading to enhanced neurological function. Additionally, LEFTY2 significantly reduced Aβ1-42 levels in Ts21 neurons and mitigated neuroinflammation.

In a co-culture environment of ADSCs and Ts21 neurons, we observed a significant increase in the proliferation of Ts21 neurons (Figure 1A,B). ELISA revealed a substantial upregulation of LEFTY2 protein in the culture medium (Figure 2C). qPCR analysis revealed no significant difference in LEFTY2 mRNA levels in ADSCs during single culture, nor in Ts21 neurons under either single or co-culture conditions. Interestingly, we found an increase in LEFTY2 mRNA levels in ADSCs specifically during co-culture, suggesting that the LEFTY2 present in the co-culture medium originates from ADSCs (Figure 2D). Subsequent Western blot analysis for LEFTY2 revealed no increase in LEFTY2 expression was observed in either cell type under single culture conditions; however, under co-culture conditions, there was an increase in LEFTY2 expression in both ADSCs and Ts21 neurons (Figure 2E). These results imply that LEFTY2 may be secreted by ADSCs during co-culture and subsequently taken up by Ts21 neurons.

Elevated TGF-β1 levels have been associated with an increased risk of AD [20], particularly in individuals carrying the APOE4 allele. The presence of APOE4 leads to the induction of TGFβ-mediated checkpoints, which dampen microglial activation and their ability to respond effectively to the neurodegenerative environment. This altered response contributes to the progression of Alzheimer’s disease, as the microglia become less efficient in clearing amyloid plaques and responding to neuronal injury [20,21]. LEFTY2, known for its inhibitory effect on TGF-β1 signaling, exerts its action by interfering with TGF-β1-induced phosphorylation of Smad2 and Smad3—key receptor-regulated Smads in the TGF-β pathway. This inhibition occurs at the level of the TGF-β receptor, preventing its downstream signaling cascade and subsequently reducing the mRNA and protein expression of TGF-β1 target genes, such as α-SMA and COL1a1 [15,22]. LEFTY2’s ability to inhibit TGF-β-induced Smad3 phosphorylation offers a potential strategy to modulate the effects of TGF-β in Alzheimer’s disease. By alleviating the suppressed microglial response caused by APOE4. Furthermore, our findings suggest that LEFTY2 binds to and downregulates APOE4, offering an additional mechanism by which LEFTY2 could benefit Alzheimer’s disease pathology.

APOE4 is a well-established genetic risk factor for AD, contributing to increased amyloid deposition and impaired function of the brain [23]. Our findings demonstrated a notable reduction in APOE4 protein expression within Ts21 neurons following co-culture with ADSCs and LEFTY2 supplementation (Figure 1J and Figure 5G). A comprehensive analysis of AD-related genes within Ts21 neurons revealed that LEFTY2 effectively downregulates APOE4 gene expression (Figure 5C). Although only a slight decrease in APP gene expression was observed (Figure 5A), supplementation with 2 nM LEFTY2 led to a significant reduction in APP protein levels (Figure 5E). This discrepancy may be attributed to temporal differences in gene and protein regulation, wherein the reduction in APP transcript levels returns to baseline while the protein levels remain reduced. In contrast, PSEN1 expression was not significantly altered at either the gene (Figure 5B) or protein level (Figure 5F) following LEFTY2 supplementation. We hypothesized a potential interaction between LEFTY2 and APOE4 protein to explain the downregulation of the expression of the APOE4 protein by LEFTY2. Surface plasmon resonance analysis confirmed a high-affinity binding between LEFTY2 and APOE4 (1.84 × 10^−9^). The lowest effective binding concentration of LEFTY2 to APOE4 was determined to be 2 nM (Figure 5H). These results confirm the ability of LEFTY2 to interact with and modulate APOE4 function. Furthermore, our findings established a direct correlation between elevated LEFTY2 concentrations and reduced APOE4 protein levels.

Cell viability assay confirmed the beneficial effects of LEFTY2 protein supplementation on Ts21 neuron growth (Figure 3A). In the brains of AD patients, nerve cells utilize SYN to produce and secrete synaptic vesicles, essential structures for storing and releasing neurotransmitters [24]. AD patients exhibit a significant decrease in SYN expression [16,24,25]. Reduction in SYN levels was closely associated with cognitive decline, as it hinders inter-neuronal information transmission, leading to memory and cognitive impairment. PSD-95, a protein abundantly present in the brain, constitutes the primary scaffold protein of postsynaptic sites in excitatory synapses and plays a pivotal role in maintaining synaptic structure and function. Excessive accumulation of amyloid protein can decrease PSD-95, reducing synaptic stability and neurotransmission [24,26]. Our study demonstrated that LEFTY2 can effectively upregulate the expression of SYN and PSD-95 proteins (Figure 3D,E). These findings collectively suggest that LEFTY2 enhances the neuronal signaling capabilities of Ts21 neurons.

By quantifying Aβ1-42 protein levels within cells, we determined that a concentration of 2 nM LEFTY2 is sufficient to attenuate amyloid protein expression (Figure 4A–C). These results collectively suggest that LEFTY2 exerts its beneficial effects on AD-affected cells through various mechanisms. The ability of LEFTY2 to modulate APOE4, APP, and Aβ1-42 expression might be the key to mitigating the impact of amyloid on neurons, although further research is required to elucidate the underlying mechanisms. LEFTY2 supplementation significantly influences neuroinflammatory markers, specifically TREM2 and MIF, which are closely associated with Tau pathology in AD. TREM2, a receptor expressed on microglia, has been recognized for its protective role in AD, enhancing microglial activity, promoting the clearance of Aβ, and attenuating Tau pathology [27,28]. The elevated levels of TREM2 observed following LEFTY2 treatment (Figure 6C) indicate that it may activate microglial responses, reducing Tau phosphorylation and potentially mitigating neurodegenerative processes.

Conversely, MIF is a pro-inflammatory cytokine elevated in AD and has been associated with increased neuroinflammation and Tau hyperphosphorylation [29]. Elevated MIF levels contribute to a pro-inflammatory state that exacerbates Tau aggregation and neuronal damage [30]. In our study, the significant reduction in MIF levels following LEFTY2 treatment (Figure 6D) indicates that LEFTY2 may effectively attenuate inflammation, thereby mitigating Tau pathology. These results suggest modulating TREM2 and MIF levels through LEFTY2 may provide a novel therapeutic strategy for addressing Tau-related neurotoxicity in AD.

Although Ts21 neurons inherently overexpress amyloid precursor protein (APP) due to the presence of an extra copy of chromosome 21, this model does not fully replicate the natural progression of Alzheimer’s disease (AD). AD is a highly complex and multifactorial disorder that develops through a series of intricate, multistep pathological processes, including amyloid-beta (Aβ) accumulation, tau pathology, neuroinflammation, and synaptic dysfunction. It is important to acknowledge that the overexpression of APP in Ts21 neurons primarily reflects APP-related pathways of AD pathogenesis. However, this model may not fully capture the molecular mechanisms underlying sporadic AD or late-onset AD, particularly in cases where APP mutations are absent or where other genetic and environmental risk factors play a significant role. Despite these limitations, Ts21 neurons provide a valuable system for studying the impact of APP overexpression in AD.

LEFTY2 protein demonstrates multiple beneficial effects during the co-culture of ADSCs and Ts21 neurons, including a reduction in amyloid protein, the promotion of nerve cell growth, and an increase in synaptic protein expression. These findings collectively suggest that LEFTY2 holds significant promise as a therapeutic agent for AD. However, LEFTY2’s inability to cross the blood-brain barrier (BBB) presents a significant challenge to its direct application in the treatment of neurodegenerative diseases. Encapsulation of LEFTY2 within exosomes may facilitate its successful traversal of the BBB, thereby enabling its therapeutic potential in neurological disorders [14]. This approach holds promise for enhancing the therapeutic efficacy of LEFTY2 in treating neurodegenerative conditions. Future research should focus on further optimizing exosome-based delivery systems to ensure the efficient and selective transport of LEFTY2 across the BBB. Additionally, investigations into the ability of LEFTY2 to alleviate memory and behavioral deficits in genetically modified animal models of AD will be crucial to advancing its therapeutic potential.

## 4. Experimental Procedures

### 4.1. Human Adipose Stem Cell Culture and Subculture

The human adipose stem cells (hADSCs) employed in this study were provided by Dr. Wei WuLi of the Hualien Tzu Chi Hospital (REC Number: IRB112-094-A). The hADSCs were cultured in Keratinocyte-SFM medium (Gibco, Waltham, MA, USA, 10724-011) supplemented with EGF Human Recombinant (Gibco, 10450-013), Bovine Pituitary Extract (Gibco, 13028-014), Ascorbic Acid 2-Phosphate (Sigma-Aldrich, St. Louis, MO, USA, 49752), N-Acetyl-L-Cysteine (Sigma-Aldrich, A8199-100G), Fetal Bovine Serum (Gibco, 26140-079), and Penicillin–Streptomycin (Gibco, 15140-122) within a humidified incubator maintained at 37 °C and 5% CO_2_. Cell growth was monitored daily under an inverted microscope, and the culture medium was changed every two days. For cell subculture, the cells were washed twice with 1× phosphate-buffered saline (PBS) (GeneDirex, Taichung, Taiwan, CC702-0500), followed by treatment with trypsin/EDTA (Gibco, 25200-072). Subsequently, the cells were incubated in an incubator at 37 °C and 5% CO_2_ for 5 min. The trypsin reaction was terminated with an equal volume of culture medium. The suspended cells were centrifuged at 1200 rpm for 3 min. Following centrifugation, the cells were resuspended in fresh culture medium and transferred to new 75 cm^2^ culture plates, each containing 10 mL of culture medium.

### 4.2. Ts21-iPSCs

The induced pluripotent stem cells overexpressing the APP gene used in this study were Ts21-iPSCs provided by Dr. Chia-Yu Chang from the Bioinnovation Center, Buddhist Tzu Chi Medical Foundation [31]. The Ts21-iPSCs were cultured in Essential 8^TM^ medium (Gibco, A1517-001) with 1% Essential 8™ supplement (Gibco, A28584-01) and 1% Penicillin–Streptomycin (Gibco, 15140-122) within a humidified incubator maintained at 37 °C and 5% CO_2_. Cell growth was monitored daily under an inverted microscope, and the culture medium was changed every two days. For cell subculture, the cells were washed twice with 1x PBS (GeneDireX, CC702-0500), followed by treatment with Accutase (Gibco, A11105-01). Subsequently, the cells were incubated at 37 °C and 5% CO_2_ for 2 min. The Accutase reaction was terminated with an equal volume of culture medium. The suspended cells were centrifuged at 1000 rpm for 2 min. Following centrifugation, the cells were resuspended in fresh culture medium, transferred to new 6 cm^2^ culture plates (Falcon, Corning, NY, USA, 353002), and pre-coated with 1% Matrigel matrix (Corning, Corning, NY, USA, 354230). Each plate contained 5 mL of culture medium.

### 4.3. Neuronal Differentiation of Ts21-Derived Induced Pluripotent Stem Cells

The culture and differentiation protocols for Ts21-derived induced pluripotent stem cells were adapted from the established literature [31]. Neuronal differentiation was performed after the cells reached 90% confluency. The cells were washed twice with 1x PBS (GeneDireX, CC702-0500), followed by treatment with Accutase (Gibco, A11105-01). Subsequently, the cells were incubated at 37 °C and 5% CO_2_ for 2 min. The Accutase reaction was terminated with an equal volume of culture medium. The suspended cells were centrifuged at 1000 rpm for 2 min. Following centrifugation, the cells were resuspended and cultured in suspension using a DMEM/F12 (Gibco, 11330-032) medium supplemented with 20% KnockOut Serum Replacement (Gibco, 10828-028), 1% L-Glutamine (Gibco, 25030-081), 1% NEAA (Gibco, 11140-050), and 1% Penicillin–Streptomycin (Gibco, 15140-122) to form the embryoid bodies. The cells were cultured in suspension.

Twenty-four hours post-plating, the culture medium was replaced with a neural induction medium, a 2:1 mixture of High-Glucose DMEM (Gibco, 11965-092), and F12 medium (Gibco, 12660-012) supplemented with 1% N-2 supplement (Gibco, 17502-048), 1% L-Glutamine (Gibco, 25030-081), 1% NEAA (Gibco, 11140-050), 1% Penicillin–Streptomycin (Gibco, 15140-122), 1 μg/mL, 0.5 μM BIO (Sigma-Aldrich, B1686), 10 μM SB431542 (Sigma-Aldrich, S4317), and 10 ng/mL FGF-2 (Sigma-Aldrich, F0291). The cells were cultured in the neural induction medium for two days.

Following a two-day culture in the neural induction medium, the medium was replaced with Neurobasal Medium (Gibco, 21103-049), supplemented with 1% B-27 supplement (Gibco, 17504-044), 1% N-2 supplement (Gibco, 17502-048), 1% L-Glutamine. (Gibco, 25030-081), 1% NEAA (Gibco, 11140-050), and 1% Penicillin–Streptomycin (Gibco, 15140-122). The cells were cultured in Neurobasal Medium for 30 days, and the culture medium was changed every two days. The cells were analyzed after a 30-day period to determine their successful differentiation into neurons exhibiting AD characteristics, following established protocols from the previous literature [31].

### 4.4. Ts21 Neurons Co-Culture with ADSCs

Six-well culture plates (Falcon, 353046) were pre-coated with a 1% Matrigel matrix (Corning, 354230). Differentiated Ts21-iPSC neurons were treated with Accutase (Gibco, A11105-01) and incubated at 37 °C and 5% CO_2_ for 2 min. The Accutase reaction was terminated with an equal volume of culture medium, and cells were gently pipetted to disperse. Differentiated Ts21-iPSC neurons were seeded at a density of 1 × 10⁶ cells per well and cultured in Neurobasal Medium for 24 h to allow differentiation into Ts21 neurons. Adipose stem cells were seeded at a density of 1 × 10^5^ cells into an 8 μm cell insert (Falcon, 353093) and cultured in Keratinocyte-SFM medium (Gibco, 10724-011) for 24 h to promote adherence. After 24 h, the Neurobasal Medium in the Ts21 neuron plate was replaced with 2 cc of fresh Neurobasal Medium. The cell inserts containing adipose stem cells were then placed on the cell culture dish containing Ts21 neurons, and 1 cc of fresh Neurobasal Medium was added to the cell inserts. Ts21 neurons were co-cultured with ADSCs for a total of 48 h.

### 4.5. Immunofluorescence Staining

Ts21-iPSCs were differentiated for 30 days. Cell culture chamber slides (08-774-25, Falcon) were pre-coated with a 1% Matrigel matrix (Corning, 354230) for 30 min before seeding differentiated Ts21-iPSC neurons. The cells were cultured in Neurobasal Medium (Gibco, 21103-049) supplemented with 1% B-27 supplement (Gibco, 17504-044), 1% N-2 supplement (Gibco, 17502-048), 1% L-Glutamine (Gibco, 25030-081), 1% NEAA (Gibco, 11140-050), and 1% Penicillin–Streptomycin (Gibco, 15140-122) for a total of 10 days. Neuronal filament staining was performed following this 10-day culture period.

Ts21 neuron cells were washed twice with 1x PBS (GeneDireX, CC702-0500). Cells were fixed with 4% paraformaldehyde for 15 min, then washed twice with 1x phosphate-buffered saline with Tween (PBS-T). Cell membranes were permeabilized with 0.1% Triton X-100 (Sigma-Aldrich, 9036-19-5) for 10 min, followed by gentle rinsing with 1x PBS-T. Cells were blocked with 5% fetal bovine serum (FBS) (Gibco, 26140-079) for 60 min, then incubated with primary antibody at 4 °C for 24 h. After washing twice with 1x PBS-T, a secondary antibody was added and incubated at room temperature for 60 min. Following another round of washing with 1x PBS-T, cell nuclei were stained with 4’,6-diamidino-2-phenylindole (Sigma-Aldrich, 28718-90-3) for visualization and photography under a fluorescence microscope. The total sample size is 3 (*n* = 3), with each experiment conducted in triplicate.

### 4.6. RT-qPCR

This method includes three parts: RNA extraction, cDNA synthesis, and RT-qPCR. Total RNA was isolated from the cells using the RNeasy Mini Kit (Qiagen, Hilden, Germany, 74104). Subsequently, cDNA was synthesized from the extracted RNA using the ReverAid First Strand cDNA Synthesis Kit (Thermo Fisher Scientific, Waltham, MA, USA, K1622). For RT-qPCR, cDNA was combined with Power SYBR Green PCR Master Mix (Thermo Fisher Scientific, 1907592), primers, and UltraPure™ DNase/RNase-Free Distilled Water (Thermo Fisher Scientific, 10977015). RT-qPCR was performed on an Applied Biosystems™ QuantStudio™ 3 Real-Time PCR System (Thermo Fisher Scientific, A28136) using the following thermal cycling conditions: 50 °C for 2 min, 95 °C for 10 min, followed by 40 cycles of denaturation at 95 °C for 10 s, annealing at 60 °C for 1 min, and extension at 95 °C for 15 s. The total sample size is 3 (*n* = 3), with each experiment conducted in triplicate.

### 4.7. Enzyme-Linked Immunosorbent Assay

ELISAs were performed on the culture medium using commercially available ELISA kits specific for LEFTY2 (Invitrogen, Waltham, MA, USA, EH298RB), Human Amyloid beta ELISA Kit, Ultrasensitive (Invitrogen, KHB3544), Human Tau (Phospho) [pT231] (Invitrogen, KHB8051), Human Tau (Phospho) [pT181] (Invitrogen, KHO0631), Human Tau (Phospho) [pS199] (Invitrogen, KHB7041), Human Tau (Total) ELISA Kit (Invitrogen, KHB0041), following the manufacturer’s instructions. For co-culture, a mixed medium collected from both compartments was used for analysis. The optical density of the developed colorimetric reaction was measured at 450 nm using a Synergy™ HTX Multi-Mode Reader (BioTek Instruments, Winooski, VT, USA). The total sample size is 3 (*n* = 3), with each experiment conducted in triplicate.

### 4.8. Western Blot

Equal amounts of protein were separated by sodium dodecyl sulfate–polyacrylamide gel electrophoresis on a 10% gel at 80 V for 30 min, followed by 160 V for 60 min. Proteins were transferred to a 0.45 μm polyvinylidene fluoride membrane (Merck Millipore, Darmstadt, Germany, IPVH00010) at 400 mA for 60 min. The membrane was blocked with 5% non-fat dry milk in Tris-buffered saline (GeneMark, Taichung, Taiwan, GB08-4) containing 0.1% Tween 20 (VWR, PT-0777-500 mL) (TBS-T) for 1 h at room temperature with gentle shaking. After three washes with TBS-T for 10 min each, the membrane was incubated with primary antibody against LEFTY2 (1:1000; Abcam, Cambridge, UK, Ab229668), APP (1:250; Invitrogen, 13-0200), APOE4 (1:1000; Cell Signaling Technology (CST), Danvers, MA, USA, 8941), PSEN1 (1:1000; Cell Signaling, 5643), Anti-beta Amyloid 1-42 antibody [mOC64] (1:1000; Abcam, Ab201060), SYN (1:1000; Abcam, Ab32127), PSD95 (1:1000; Abcam, Ab18258) at 4 °C overnight with shaking. Subsequent washes with TBS-T were followed by incubation with secondary antibody: Goat anti-mouse IgG (1:5000; Croyez Biosciences, Chennai, India, C04001) or Goat anti-rabbit IgG (1:5000; Croyez, C04003) at room temperature for 1 h with shaking. After another three washes with TBS-T, the membrane was developed using ECLong (GeneDireX, SM801-0500) according to the manufacturer’s instructions. The chemiluminescent signal for the protein of interest was captured and quantified using iBright™ FL1500 Imaging System (Invitrogen, A44115).

Following chemiluminescent signal capture, the membrane was stripped of bound antibodies using ReBlot Plus Strong Antibody Stripping Solution (Merck, Darmstadt, Germany, 2504) for 40 min at 80 rpm. After three washes with TBS-T for 10 min each, the membrane was blocked with 5% non-fat dry milk in Tris-buffered saline (GeneMark, GB08-4) containing 0.1% Tween 20 (VWR, PT-0777-500 mL) (TBS-T) for 1 h at room temperature with gentle shaking. The membrane was then incubated with a primary antibody against beta-actin (1:5000; Invitrogen, MA1-140) at 4 °C overnight with shaking. Subsequent washes with TBS-T were followed by incubation with secondary antibody Goat anti-mouse IgG (1:5000; Croyez, C04001) at room temperature for 1 h with shaking. After another three washes with TBS-T, the membrane was developed using ECLong (GeneDireX, SM801-0500) according to the manufacturer’s instructions. The chemiluminescent signal for beta-actin was captured and quantified using the iBright™ FL1500 Imaging System (Invitrogen, A44115). For the negative control, cell lysates from cells that do not express the target protein were used. The corresponding negative control bands are provided as supplementary figures. The total sample size is 4 (*n* = 4) for APOEE4 and LEFTY2 under co-culture conditions and sample size of 3 (*n* = 3) for other groups, with each experiment conducted in triplicate.

### 4.9. Surface Plasmon Resonance

This method includes two parts: immobilization of human APOE4 on a Sensor Chip CM5 surface and kinetic measurement. Human APOE4 was covalently immobilized on the surface of a Sensor Chip CM5 via amine coupling chemistry. A freshly prepared 1:1 mixture of 100 mM NHS and 400 mM EDC was injected into flow cell two at a flow rate of 10 µL/min for 420 s to activate the dextran matrix on flow cell two of a Sensor Chip CM5. Human APOE4 was diluted in immobilization buffer (10 mM sodium acetate, pH 4.5) to a final concentration of 10 µg/mL. This diluted ligand was injected into the activated flow cell two at a flow rate of 10 µL/min to reach immobilization levels at 207.5 response units. To deactivate the excess reactive groups, 1 M methanolamine was injected into flow cell two at a 10 µL/min flow rate for 420 s.

A kinetic measurement assay was performed by using the Kinetic/Affinity wizard. The flow path was 2-1, as the ligand, human ApoE4, was immobilized in flow cell 2, and flow cell 1 acted as a reference. A series of concentrations of human LEFTY2 were injected over the reference and the ligand surfaces consecutively as the association phase, with short dissociation phases in between by injecting a running buffer. Then, the regeneration solution was injected as the regeneration phase. All the procedures were conducted at 25 °C. All the resulting data were fitted to a 1:1 binding model using Biacore T200 Evaluation Software, version 3. The total sample size is 3 (*n* = 3), with each experiment conducted in triplicate.

### 4.10. Protein Digestion Using Filter-Aided Sample Preparation Method

Culture medium was harvested and processed by filter-aided sample preparation [32] method for MS analysis. Alkylation of samples was performed by adding iodoacetamide (Thermo Fisher Scientific, 144-48-9) to a final concentration of 50 mM and incubated in the dark for 30 min. Then, 10 μg of reduced and alkylated protein complexes were loaded onto a Microcon YM-30 cut-off filter (Millipore, MRCF0R030), followed by repeated washes (at least 5 times) with 300 μL of 8 M urea buffer and centrifuged at 34,000× *g* for 15 min. Next, we performed trypsin digestions directly on the filter in overnight reactions at room temperature on a shaking platform. Trypsin solution (Thermo Fisher Scientific, 9002-07-7) was added in a ratio of 1:100 (*w*/*w*) in 50 mM ammonium bicarbonate (Thermo Fisher Scientific, 1066-33-7). Peptides were collected by adding 50 μL of 0.5 M NaCl (Thermo Fisher Scientific, 7647-14-5) to the filter and centrifuged at 14,000× *g* for 10 min to ensure the elution of most tryptic peptides. Finally, the collected peptide samples were processed with a Zip-Tip C18 reversed phase pipette tip (Millipore, ZTC18S096) for desalting, concentration, and cleaning according to manufacturer protocol. The samples were resuspended in 100 μL of 0.1% CF3COOH (TFA) (Thermo Fisher Scientific, 76-05-1) and sonicated in a water bath for 1 min. The total sample size is 3 (*n* = 3), with each experiment conducted in triplicate.

### 4.11. MS/MS Protein Identification and Quantification

For label-free, relative, quantitative analysis, each sample was analyzed by nano-LC−MS/MS. For each run, 1 μg of the sample was injected on a 75 μm × 250 mm, reversed-phase C18 Acclaim PepMap column (Thermo Fisher Scientific, REF) with 3 μm particles using an UltiMate 3000 RSLC Nano System (Thermo Fisher Scientific, ULTIM3000RSLCNANO). Chromatography solvents were water (A) and 80% acetonitrile (Thermo Fisher Scientific, 75-05-8) (B), both with 0.1% formic acid (Thermo Fisher Scientific, 64-18-6). Peptides were eluted from the column with the following gradient. The gradient was run as follows: 0–8% B for 10 min, then 50% B at 97 min, and 100% B at 110 min. Peptides eluting from the column were analyzed by data-dependent MS/MS on a Q-Exactive Orbitrap mass spectrometer. A top 10 method was used to acquire data. In brief, the instrument settings were as follows: resolution was set to 70,000 for MS scans and 17,500 for the data-dependent MS/MS scans to increase speed. The MS automatic gain control (AGC) target was set to 106 counts, while the MS/MS AGC target was set to 105. The MS scan range was from 300 to 2000 m/z. MS scans were recorded in profile mode, while the MS/MS was recorded in centroid mode to reduce data file size. Dynamic exclusion was set to a repeat count of 1 with a 25 s duration. Following LC−MS/MS acquisition, the data were analyzed using Proteome Discoverer 2.4 against the UniProt Human database (6/23/2013, 20 209 sequences) by SEQUEST at a false discovery cut-off ≤ 1%. The mass tolerances were 20 ppm for precursor and 0.5 Da for product ions. Two missed cleavages were allowed. The search engine set cysteine carbamidomethylation as a fixed modification, N-terminal acetylation, and methionine oxidation as variable modifications. The quantification was performed based on the peak intensities of the report ions of the unique peptides in the MS/MS Spectra. The total sample size is 3 (*n* = 3), with each experiment conducted in triplicate.

### 4.12. Statistical Analysis

Statistical analyses were performed using unpaired Student’s t-tests for comparisons between two groups and one-way ANOVA followed by Tukey’s post hoc test for multiple group comparisons. Data are presented as the mean ± standard error of the mean (SEM). A *p*-value of less than 0.05 was considered statistically significant and denoted by an asterisk (*). Sample sizes for each experimental group are indicated in the figure legends.

## Data Availability

The original contributions presented in this study are included in the article/Supplementary Material. Further inquiries can be directed to the corresponding author or lead contact.

## Supplementary Materials

The following supporting information can be downloaded at: https://www.mdpi.com/article/10.3390/ijms26073382/s1.
