# Correction: Li et al. The Therapeutic Potential of ADSC-Secreted LEFTY2 in Treating Alzheimer’s Disease. *Int. J. Mol. Sci.* 2025, *26*, 3382

**DOI:** 10.3390/ijms26157351

**Published:** 2025-07-30

**Authors:** Wei-Wu Li, Hsueh-Hui Yang, Tzyy-Wen Chiou, Peng-Yeong Woon, Yue-Xuan Xu, Cynthia Tjandra, Ivan Wijaya, Horng-Jyh Harn, Shinn-Zong Lin

**Affiliations:** 1 Bioinnovation Center, Buddhist Tzu Chi Medical Foundation, Hualien 97002, Taiwan; dionysus2316@gmail.com (W.-W.L.); xuan01101994@gmail.com (Y.-X.X.); 2 Department of Medical Research, Hualien Tzu Chi Hospital, Buddhist Tzu Chi Medical Foundation, Hualien 97002, Taiwan; hhyang@tzuchi.com.tw; 3 Department of Life Science, National Dong Hwa University, Hualien 974301, Taiwan; twchiou@mail.ndhu.edu.tw; 4 Department of Molecular Biology and Human Genetics, Tzu Chi University, Hualien 97004, Taiwan; woon07@gms.tcu.edu.tw (P.-Y.W.); 112727103@gms.tcu.edu.tw (C.T.); ivanwjy74@gmail.com (I.W.); 5 Department of Pathology, Hualien Tzu Chi Hospital, Hualien 97002, Taiwan; 6 Department of Neurology, Hualien Tzu Chi Hospital, Hualien 97002, Taiwan

## Missing Funding

In the original publication [1], the funder Gwo Xi Stem Cell Applied Technology Co., Ltd. (grant number: IAC-RD-21-002) was not included. The corrected Funding appears below:

**Funding:** This research was supported by the National Science and Technology Council, R.O.C (grant number NSTC 113-2314-B-303-005) and Gwo Xi Stem Cell Applied Technology Co., Ltd. (grant number: IAC-RD-21-002).

## Conflicts of Interest Correction

The authors would like to modify the Conflict of interest Section of the following published paper [1]. The changes are below.

**Conflicts of Interest:** The authors declare that this study received funding from Gwo Xi Stem Cell Applied Technology Co., Ltd. (grant number: IAC-RD-21-002). The funder was not involved in the study design, collection, analysis, interpretation of data, the writing of this article or the decision to submit it for publication.

The authors state that the scientific conclusions are unaffected. This correction was approved by the Academic Editor. The original publication has also been updated.
