# Supplementary material for: The Therapeutic Potential of ADSC-Secreted LEFTY2 in Treating Alzheimer’s Disease
Source: Int J Mol Sci. 2025 Apr 4;26(7):3382. doi: 10.3390/ijms26073382 (PMC11990000; doi:10.3390/ijms26073382)
Supplement: Supplementary file 1 [file ijms-26-03382-s001.zip › ijms-3528077-supplementary.pdf]

Supplemental Information

The Therapeutic Potential of ADSC-secreted LEFTY2 in treating Alzheimer's Disease

Wei WuLi, Hsueh-Hui Yang, Tzyy-Wen Chiou, Peng Yeong Woon, Yue-Xuan Xu, Cynthia Tjandra, Ivan Wijaya, Horng-Jyh Harn, Shinn- Zong Lin

Supplementary Methods

Electrophysiological Recording of iPSC-Derived Cells

Whole-cell patch-clamp recordings were performed at room temperature. Coverslips containing the cultured cells were transferred to a recording chamber, which is continuously perfused with oxygenated artificial cerebrospinal fluid (aCSF; 95% O_2, 5% CO_2). Patch-clamp electrodes were fabricated from borosilicate capillary glass (outer diameter: 1.5 mm, inner diameter: 1.0 mm) (World Precision Instruments, PG52151-4) using a Sutter P-97 micropipette puller (Sutter Instrument, Novato, CA). The electrode tips were fire-polished with an MF-830 microforge (Narishige, Tokyo, Japan) to optimize resistance.

Once filled with intracellular pipette solution, electrode impedance in aCSF will range from 6 to 15 MΩ. Junction potential will be zeroed, and series resistance will not be compensated. After establishing contact with the cell membrane, a high-resistance seal (≥2 GΩ, typically 5–10 GΩ) will be achieved via gentle suction. Whole-cell currents will be recorded using an Axoclamp 200B amplifier (Axon Instruments, Union City, CA). Voltage-clamp mode will be employed to assess ion channel- and receptor-mediated currents as well as intrinsic membrane properties. Cells will be classified as neurons only if they exhibit fast-inactivating inward currents. During recording, the membrane potential will be held at -60 mV, followed by step depolarizations from -80 mV to +40 mV in 10 mV increments (400 ms duration).

Supplementary Figure 1 and legend

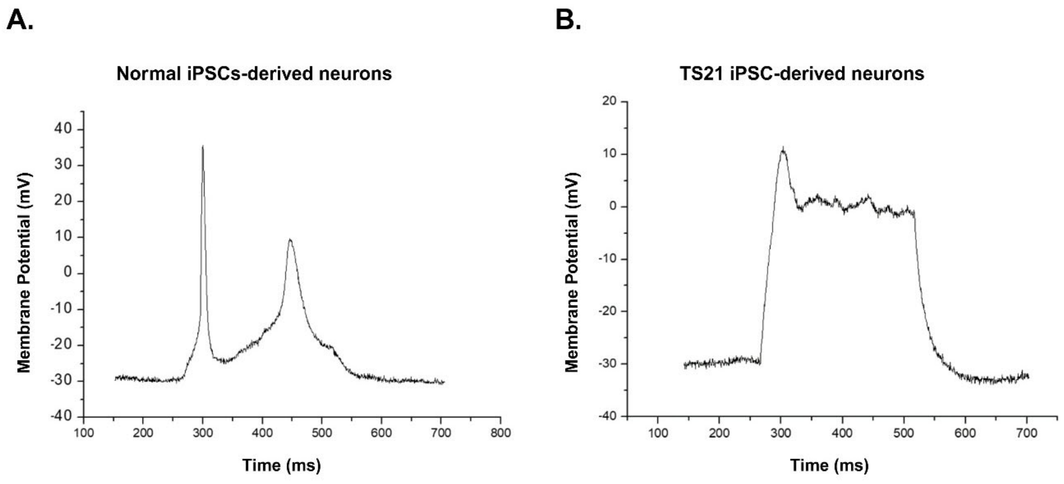

Figure S1. Patch-clamp analysis of neurons derived from normal iPSCs (left) and Ts21 iPSCs (right). The analysis revealed significant differences in the electrophysiological properties of Ts21 neurons, including prolonged depolarization and delayed repolarization, compared to neurons derived from normal iPSCs.

Supplementary Figure 2 and legend

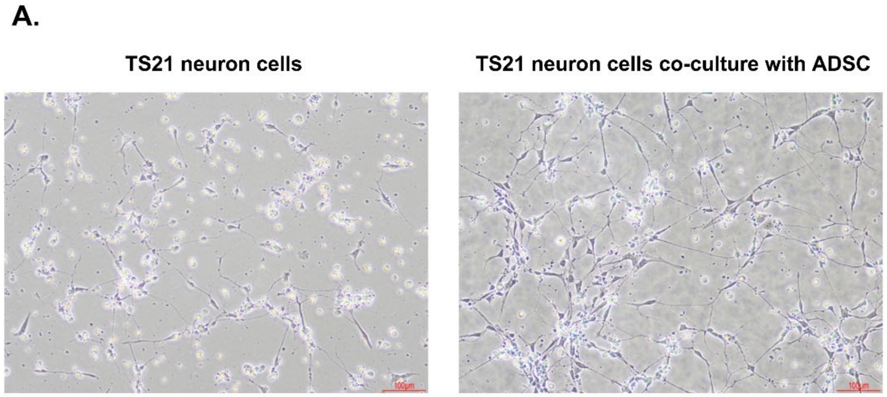

Figure S2. Microscopic image of representative Ts21 neuron cells (left) compared to Ts21 neuron cells co-cultured with ADSCs (right). Scale bar: 100 μm.

Supplementary Figure 3 and legend

A.

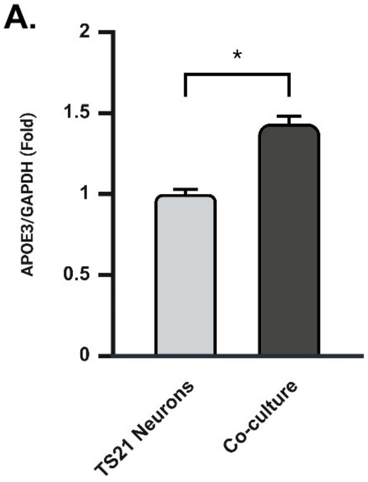

Figure S3. APOE3 gene expression in Ts21 neurons in single-culture and co-culture conditions. APOE3 expression was significantly increased in Ts21 neurons following co-culture (Ts21 neurons: 1.00 ± 0.03; Co-culture: 1.44 ± 0.5), n = 3 for each group. Values are mean ± SEM; * $p < 0.05$. ** $p < 0.01$ by Student's t-tests.

Supplementary Figure 4 and legend

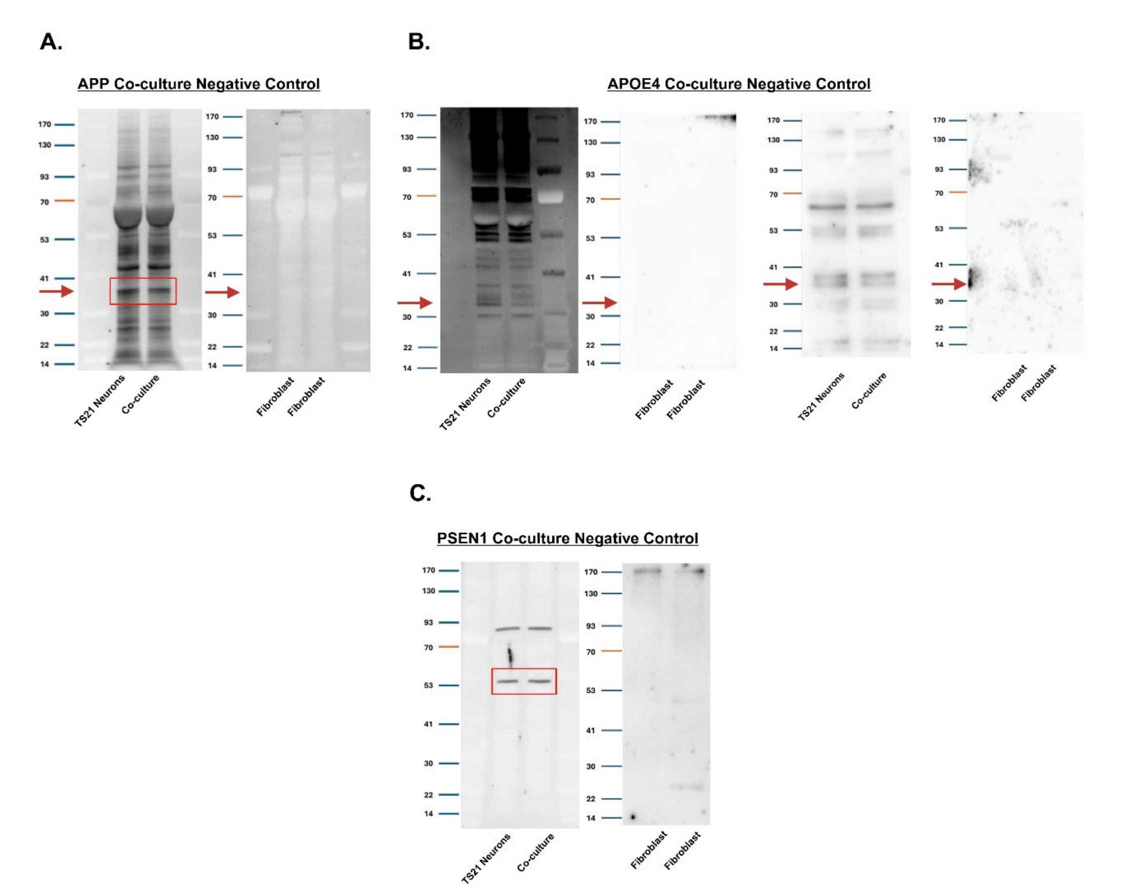

Figure S4. Representative bands for normal (left) and negative control (right) for (A) APP, (B) APOE4, and (C) PSEN1.

Supplementary Figure 5 and legend

A.

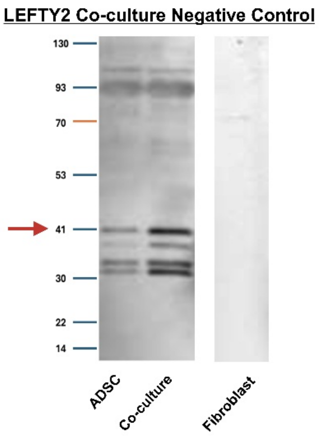

Figure S5. Representative bands for normal (left) and negative control (right) for LEFTY2.

Supplementary Figure 6 and legend

A.

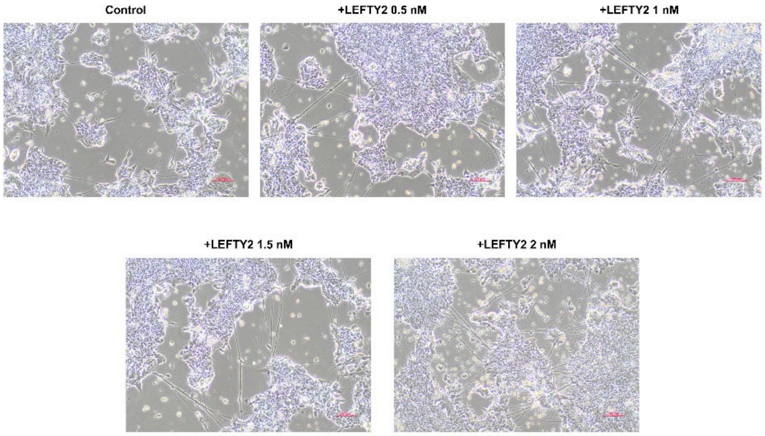

B.

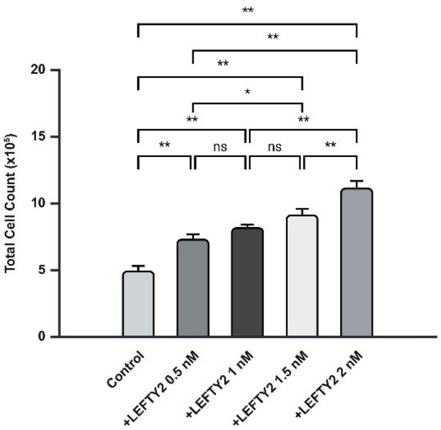

Figure S6. Cell morphology and cell viability analysis after LEFTY2 supplementation. (A) Microscopic image of a representative Ts21 neuron cell supplemented with 0 nM (top-left), 0.5 nM (top-middle). 1 nM (top-right), 1.5 nM (bottom left), and 2 nM (bottom-right) of LEFTY2. Scale bar: 100 μm. **(B)** The average number of Ts21 neurons following supplementation of 0 nM, 0.5 nM, 1 nM, 1.5 nM, and 2 nM of LEFTY2. (Control = 4.97 ± 0.35, 0.5 nM LEFTY2 = 7.37 ± 0.34, 1 nM LEFTY2 = 8.23 ± 0.19, 1.5 nM LEFTY2 = 9.18 ± 0.42, 2 nM LEFTY2 = 11.19 ± 0.49), n=3 for each group. Values are mean ± SEM; * $p < 0.05$. ** $p < 0.01$ by one-way ANOVA followed by Tukey post-hoc test.

Supplementary Figure 7 and legend

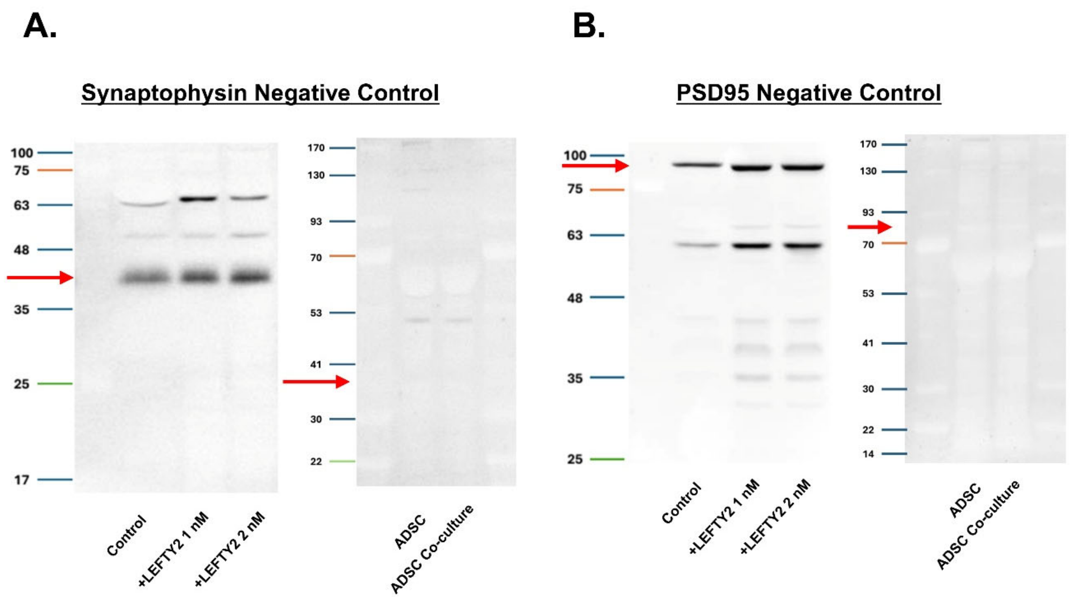

Figure S7. Representative bands for normal (left) and negative control (right) for (A) Synaptophysin and (B) PSD95.

Supplementary Figure 8 and legend

A.

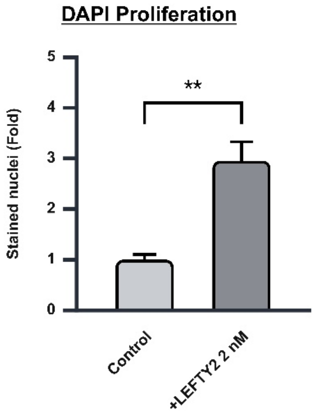

Figure S8. Quantification of DAPI-stained nuclei. The number of DAPI-stained nuclei per field was counted, revealing a significant increase in cell number following supplementation with 2 nM LEFTY2 (Control: 1.00 ± 0.11; +LEFTY2 2 nM: 2.95 ± 0.38). n = 3 for each group. Values are mean ± SEM; * $p < 0.05$. ** $p < 0.01$ by Student's t-tests.

Supplementary Figure 9 and legend

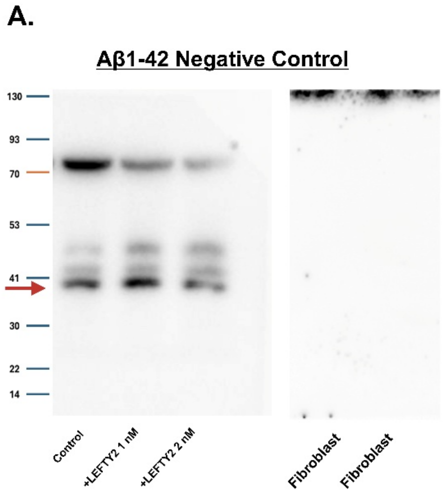

Figure S5. Representative bands for normal (left) and negative control (right) for Amyloid beta 1-42.

Supplementary Figure 10 and legend

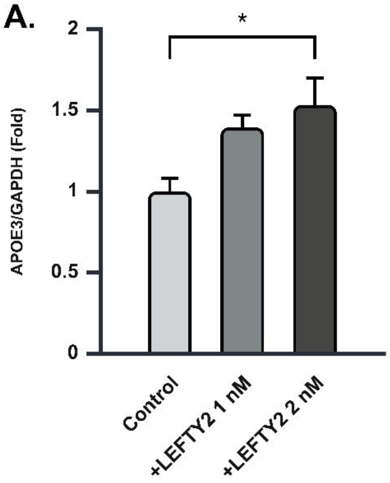

Figure S10. APOE3 gene expression in Ts21 neurons following LEFTY2 supplementation. APOE3 expression was significantly increased in Ts21 neurons following 2 nM of LEFTY2 supplementation (Control= 1.00 ± 0.08; 1 nM LEFTY2 = 1.39 ± 0.08; 2 nM LEFTY2 = 1.53 ± 0.17), n=3 for each group. Values are mean ± SEM; * $p < 0.05$. ** $p < 0.01$ by one-way ANOVA followed by Tukey post-hoc test.

Supplementary Figure 11 and legend

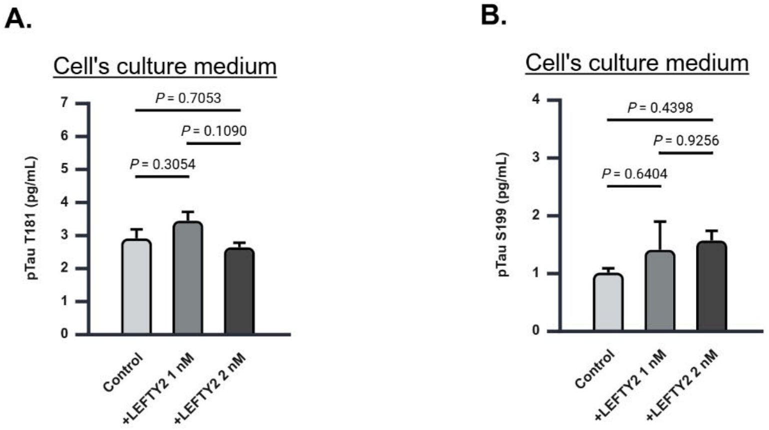

Figure S11. Phosphorylated Tau-181 and Phosphorylated Tau-199 levels after LEFTY2 supplementation. (A) Phosphorylated Tau-181 and **(B)** Phosphorylated Tau-199 levels in Ts21 neuron culture medium following 0 nM, 1 nM, and 2 nM of LEFTY2 supplementation evaluated using ELISA. No significant reduction in both pTau-181 (control = 2.89 ± 0.27, 1 nM LEFTY2 = 3.42 ± 0.26, 2 nM LEFTY2 = 2.62 ± 0.14) and pTau-199 (control = 1.00 ± 0.08, 1 nM LEFTY2 = 1.39 ± 0.49, 2 nM LEFTY2 = 1.56 ± 0.17) in the culture medium following supplementation with LEFTY2, n=3 for each group. Values are mean ± SEM; * $p < 0.05$. ** $p < 0.01$ by one-way ANOVA followed by Tukey post-hoc test.

Table S1. Overview of the primers used. All the primers were ordered from Oligo Farm. Abbreviations: Glyceraldehyde 3-phosphate dehydrogenase (GAPDH), left-right determination factor 2 (LEFTY2), amyloid-beta precursor protein (APP), presenilin 1 (PSEN1), and Apolipoprotein E4 (APOE4).

**Gene**	**Protein**	**Primer Name**	**Sequence (5' to 3')**
GAPDH	GAPDH	GAPDH Forward	CCC CAC TTG ATT TTG GAG GGA
		GAPDH Reverse	AGG GCT GCT TTT AAC TCT GGT
LEFTY2	LEFTY2	LEFTY2 Forward	TTG AGC CCT CTA ACT GAA CG
		LEFTY2 Reverse	CCA GCA TTT CCT ACT AGA GC
APP	APP	APP Forward	ATG AGC TGC TTC AGA AAG AGC
		APP Reverse	TTC GTT TTC TGT GTT GGC TGG C
PSEN1	PSEN1	PSEN1 Forward	GTG GAC TAC ATT ACT GTT GC
		PSEN1 Reverse	AAA TCA CAG CCA AGA TGA GC
APOE4	APOE4	APOE4 Forward	CGC GGA CAT GGA GGA CGT GC
		APOE4 Reverse	GCC TGG TAC ACT GCC AGG CG
APOE3	APOE3	APOE3 Forward	CGC GGA CAT GGAGGA CGT GT
		APOE3 Reverse	GCC TGG TAC ACT GCC AGG CG
